# Investigating changes in serum metabolome and urinary endocrine disrupting chemicals in cats with hyperthyroidism

**DOI:** 10.1007/s11259-026-11169-5

**Published:** 2026-03-23

**Authors:** Ayelet Ziv-Gal, Megan Mahoney, Nicolas Lopez-Villalobos, Arnon Gal

**Affiliations:** 1 Department of Comparative Biosciences, College of Veterinary Medicine, University of Illinois at Urbana-Champaign, Champaign, IL USA; 2 School of Agriculture and Environment, Massey University, Palmerston North, New Zealand; 3 Department of Veterinary Clinical Medicine, College of Veterinary Medicine, University of Illinois at Urbana-Champaign, Champaign, IL USA

**Keywords:** Thyrotoxicosis, Felis catus, Phthalate, Paraben, Untargeted mass spectrometry

## Abstract

Domestic cats share indoor environments with humans and are exposed to endocrine-disrupting chemicals (EDCs) from both household sources and cat-specific products capable of disrupting thyroid hormone signaling. The prevalence of feline hyperthyroidism (FHT) continues to rise, and while some EDCs have been implicated in its etiopathogenesis, the metabolic consequences of FHT are unknown. Here, we tested whether hyperthyroid cats exhibit altered systemic metabolomic signatures that are associated with phthalate and paraben urinary levels, compared with healthy controls. Thirty-five pet cats were enrolled (16 FHT, 19 controls). Serum samples were subjected to untargeted liquid chromatography mass spectrometry metabolomics and urine paraben and phthalates metabolites were quantified by liquid chromatography-tandem mass spectrometry. Forty-six serum metabolites and three urinary EDCs differed between groups (adjusted *p* < 0.05). Lipid metabolism pathways were enriched (16/74 significant; Fisher’s *p* = 0.02; False Discovery Rate-adjusted *p* = 0.16). Key serum differences included lower creatinine, linoleic acid, and 1-oleoyl-sn-glycerophosphoethanolamine in FHT. Urinary mono-isobutyl phthalate, ethylparaben, and propylparaben were higher in FHT (fold change 2.58, 3.30 and 2.07). Multivariable analyses separated groups; Weighted Sub-Network Analysis highlighted modules tied to tryptophan pathways, lipid homeostasis, and xenobiotic processing. Partial Least Squares captured 91% of response variance in two factors, with high-Variable Importance in Projection contributors including vitamin K1, 2-hydroxybenzothiazole, a sphingolipid long-chain base, and L-cysteine-glutathione disulfide. A Random Forest classifier achieved a 9.38% out-of-bag error and prioritized sphingoid bases and creatinine. Hyperthyroid cats had perturbed serum lipid-metabolite levels and higher urinary phthalate and paraben biomarker levels. These integrated data support an EDC-associated metabolomic signature in FHT and motivate longitudinal and mechanistic studies to clarify causality and inform prevention.

**Supplementary Information:**

The online version contains supplementary material available at 10.1007/s11259-026-11169-5.

## Highlights

- Hyperthyroid cats have higher urinary levels of phthalate and parabens
- Hyperthyroid cats have pathway-specific alterations in fatty acid metabolism
- Hyperthyroid cats have pathway-specific alterations in steroid hormones and amino acids
- Hyperthyroidism is linked to lipid imbalance, tryptophan and xenobiotic metabolism
- Sphingolipid bases are suggested as potential predictors of thyroid status

**Graphical abstract:**

**Supplementary Information:**

The online version contains supplementary material available at 10.1007/s11259-026-11169-5.

## Graphical abstract

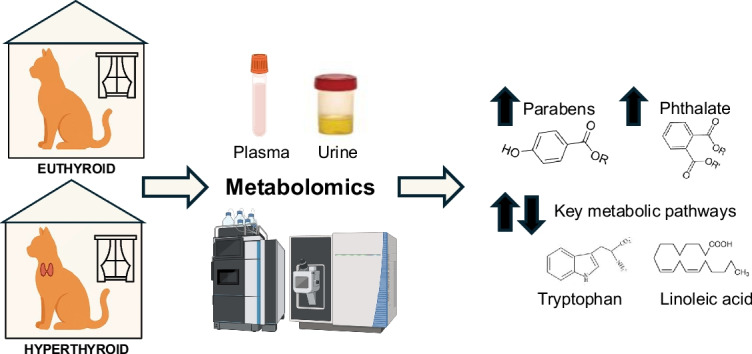

## Introduction

Feline hyperthyroidism (FHT) is now recognized as the most common endocrinopathy in mature adults to senior cats (AAHA 2021), resulting from autonomous overproduction of thyroid hormones by the thyroid gland (O'Neill et al. 2023; Peterson 2012; Vaske et al. 2014). In the vast majority of cases, FHT is caused by benign adenomatous hyperplasia of the thyroid (often affecting both lobes), whereas malignant thyroid carcinoma accounts for only a small minority (Peterson and Broome 2015; Peterson et al. 1983; Turrel et al. 1988). Since the first description of this syndrome in 1979, the reported prevalence of FHT has risen dramatically worldwide with time. Large laboratory- and practice-based datasets published within the last 5 years report feline hyperthyroidism prevalences on the order of ~ 2–6% overall (with higher values in older cats and in screened senior cohorts) (Kokkinaki et al. 2025; Miceli et al. 2023; Mortier et al. 2024; Perez Dominguez et al. 2024). Senior cats are especially at risk, with one United Kingdom study finding nearly 9% of cats over 10 years old to be hyperthyroid (Stephens et al. 2014). Clinically, affected cats often present with weight loss, polyphagia, hyperactivity, and other signs of thyrotoxicosis (Carney et al. 2016; Peterson 1984). Improved owner awareness and veterinary screening have led to earlier diagnosis and generally milder clinical manifestations today than when FHT was first recognized (Broussard et al. 1995). However, increased longevity of pet cats and heightened vigilance alone do not fully explain the rise in FHT cases over recent decades. The etiopathogenesis remains incompletely understood and is likely multifactorial (Vaske et al. 2014), with growing concern that environmental factors may contribute to this feline disease.

One prominent hypothesis is that chronic exposure to environmental endocrine-disrupting chemicals (EDCs) plays a role in the development of FHT (Chow et al. 2015; Guo et al. 2016; Nomiyama et al. 2022; Norrgran et al. 2012, 2015; Peterson 2012, 2014; Poutasse et al. 2019; Wang et al. 2018). Pet cats share indoor environments with humans and are exposed to many of the same household chemicals, including flame retardants, plasticizers, and preservatives that can interfere with thyroid hormone pathways (Ma et al. 2022; Norrgran Engdahl et al. 2017; Pocar et al. 2023; Walter et al. 2017). Brominated flame retardants, such as polybrominated diphenyl ethers (PBDEs) used in furniture and electronics, were among the first contaminants suspected in FHT’s etiology after researchers observed that the upsurge of FHT in the late twentieth century coincided with widespread PBDEs use (Holzworth et al. 1980; Nomiyama et al. 2022). Indeed, subsequent studies have shown that indoor cats tend to accumulate high levels of PBDEs and a positive correlation was noted between PBDEs and FHT risk (Norrgran Engdahl et al. 2017; Walter et al. 2017; Wang et al. 2018).

Other EDCs, particularly phthalates and parabens, are also pervasive in the indoor environment and thus may potentially contribute to FHT. Phthalates are used as plasticizers in plastics, vinyl flooring, various consumer products and some pet foods; parabens are used as antimicrobial preservatives in cosmetics, pharmaceuticals, food items and beverages, and some pet foods. Not surprisingly, measurable levels of various paraben and phthalate metabolites have been detected in feline blood, urine, feces, and commercial pet foods (Braouezec et al. 2016; Karthikraj et al. 2018; Li and Kannan 2024). These EDCs may alter thyroid hormone signaling as reported in a few mammalian species (Pearce 2024; Street et al. 2024). Yet, it remains unclear whether exposure to parabens and phthalates contributes to the pathogenesis of FHT by disrupting thyroid homeostasis.

Given the systemic effects of thyroid hormones, a systems-level approach is valuable for characterizing the biological perturbations in FHT and for detecting signatures of environmental influences. Untargeted metabolomics has emerged as a powerful discovery tool to profile global metabolic changes associated with disease states (Chen et al. 2022). In feline medicine, untargeted metabolomics has only recently been applied to FHT. A 2024 study reported that hyperthyroid cats have a distinct serum metabolome compared to healthy controls, with 277 metabolites significantly dysregulated (70 higher, 207 lower) in the hyperthyroid state (Bechtold et al. 2024). Notably, many of these metabolite perturbations (including deficits in carnitine and alpha-tocopherol) persisted even after the cats were rendered euthyroid by treatment (Bechtold et al. 2024). These results suggest that FHT involves widespread systemic metabolic derangements, some of which may represent either long-lasting consequences of thyrotoxicosis or underlying predisposing factors.

Despite growing evidence linking EDC exposure and metabolic disturbances to FHT, critical knowledge gaps remain. In the current study we tested the hypothesis that hyperthyroid cats exhibit distinct metabolomic signatures that are associated with higher urinary levels of phthalates and parabens. We aimed to (1) use untargeted metabolomics to identify key serum metabolic perturbations associated with FHT and (2) examine associations between these metabolomic features and urinary levels of selected parabens and phthalate metabolites in cats.

## Materials and methods

### Study design

We prospectively screened and enrolled 19 euthyroid controls and 16 untreated hyperthyroid cats. All cats belonged to staff, students, faculty, and clients of the Veterinary Teaching Hospital (VTH) at the University of Illinois at Urbana-Champaign and were enrolled following receipt of signed owner consent. A board-certified Small Animal Internal Medicine Specialist at the VTH (AG) examined all candidates for study enrollment. Hyperthyroid cats were included if history, physical examination, laboratory results, and serum total thyroxine (TT4) concentrations were consistent with FHT (TT4 > 49 nmol/L [reference range 17-49 nmol/L]). Hyperthyroid cats were excluded if they had received any anti-thyroidal medication within 14 days before enrollment and/or were consuming a low-iodine diet. Euthyroid control cats were included if history, physical examination, laboratory results, and serum TT4 concentrations were consistent with euthyroidism (TT4 within reference range 17-49 nmol/L). Cats of either group were excluded if they had evidence of advanced chronic kidney disease (CKD; serum creatinine > 2.9 mg/dL, International Renal Interest Society [IRIS] Stage 3 or higher) as advanced CKD may substantially alter urinary metabolite excretion and serum metabolite concentrations, which could confound metabolomic interpretation. Mild CKD (IRIS Stage 1-2) was not an exclusion criterion as it is common in senior cats (Marino et al. 2014). While CKD can be associated with metabolomic differences even in early stages, these changes are stage-dependent and more pronounced with advanced disease; therefore, we allowed IRIS stage 1-2 to preserve external validity and minimize unnecessary exclusion of typical senior cats (Nealon et al. 2024). Other concurrent endocrine diseases (e.g., diabetes mellitus, hyperadrenocorticism) and acute illness were also exclusion criteria for both groups. By design, we enrolled approximate age-matched euthyroid controls to serve as a clinically relevant comparison group, as FHT predominantly affects older cats. This age-matched approach controls for age-related metabolic changes and enables the detection of disease-specific metabolic alterations independent of aging effects. Samples were collected after overnight fasting via jugular venipuncture (serum) and ultrasound-guided cystocentesis (urine). Complete blood counts, serum biochemistry, urinalyses, and serum TT4 concentrations were measured at the Clinical Pathology Laboratory, College of Veterinary Medicine, University of Illinois at Urbana-Champaign, at the time of enrollment. Serum and urine samples designated for metabolomic analysis were immediately centrifuged, aliquoted, and stored at -80 °C. All samples were submitted in a single batch to the metabolomic core services at the University of Illinois within 12 months of collection. All procedures were performed in compliance with relevant laws and institutional guidelines after receipt of owners’ informed consent and had been approved by the University of Illinois Institutional Animal Care and Use Committee (IACUC protocol number 20012).

### Measurements of EDC levels in urine samples

Urinary EDCs were quantified using a targeted liquid chromatography-tandem mass spectrometry (LC-MS/MS) method at the Metabolomics Lab, Roy J. Carver Biotechnology Center, the University of Illinois at Urbana-Champaign. In brief, 200 µL urine samples were spiked with 10 mM ammonium formate, a mixture of deuterated internal standards, β‑glucuronidase, and 4‑methylumbelliferone glucuronide. Spiked samples were incubated at 37 °C for 90 min to hydrolyze conjugated analytes. After centrifugation, the supernatant was diluted with water and cleaned up via solid-phase extraction using Phenomenex Strata‑X cartridges. The eluent was dried and reconstituted in 60% methanol prior to analysis. Chromatographic separation was achieved on a Gemini C6‑phenyl column with a gradient using 0.1% formic acid in water (mobile phase A) and 0.1% formic acid in acetonitrile (mobile phase B). The liquid chromatography system operated at a flow rate of 0.25 mL/min with a 10 µL injection volume, and detection was performed on a Sciex 6500 + Triple Quad mass spectrometer in negative electrospray ionization mode. Quantitation was conducted using multiple reaction monitoring for various parabens and phthalates with their respective deuterated analogs, and β‑glucuronidase activity was monitored via 4‑methylumbelliferone. The following parabens and phthalate metabolites were measured: methyl-, ethyl-, propyl-, and butyl- paraben, mono-(2-ethyl-5-hydroxyhexyl) phthalate (MEHHP), mono-2-ethylhexyl phthalate (MEHP), mono-(2-ethyl-5-oxohexyl) phthalate (MEOHP), mono-(2-ethyl-5-carboxypentyl) phthalate (MECPP), mono-isobutyl phthalate (MiBP), mono-methyl phthalate (MMP), mono-benzyl phthalate (MBzP), mono (3-carboxypropyl) phthalate (MCPP), monoethyl phthalate (MEP), and monobutyl phthalate (MBP). These parabens and phthalate metabolites were selected because they represent the most commonly used parabens and the major urinary metabolites of widely used phthalates DMP, DEP, DEHP, DBP, DiBP, and BBzP (Panagiotou et al. 2021; Wei et al. 2021).

The limit of quantitation (LOQ) values per chemical are detailed in Table 1. In cases in which values were lower than (LOQ) of the assay, a value of LOQ/square root of 2 was assigned to the sample (Finkelstein and Verma 2001). Additionally, urine creatinine concentration was determined per sample to account for differences in urine dilution at the time of sampling. Urine creatinine was measured at the Clinical Pathology Laboratory, College of Veterinary Medicine, the University of Illinois at Urbana-Champaign on AU680 analyzer, Beckman Coulter. The creatinine adjusted values were then statistically analyzed as individual parabens, individual phthalate metabolites, sum paraben, and sum phthalate metabolites. Additionally, we calculated molar sums for selected metabolites by dividing the metabolites’ creatinine-normalized concentrations by their molar mass to ensure that each metabolite contributes to the summary measure on a common chemical scale (moles) rather than on a mass scale. Specifically, we generated the ‘sum PCP’ variable by adding the metabolite molar concentrations of MBP (MW 222 µg/µmol) and MEP (MW 194 µg/µmol) for the evaluation of phthalate exposure from pharmaceutical agents, shampoos, conditioners, and other personal care products (Braun et al. 2014; Kobrosly et al. 2012; Schettler 2006). We generated the ‘sum DEHP’ variable by adding the metabolite molar concentrations of MECPP (MW 308 µg/µmol), MEHHP (MW 294 µg/µmol), MEOHP (MW 292 µg/µmol), and MEHP (MW 278 µg/µmol), for the estimation of phthalate exposure from DEHP-containing products such as polyvinylchloride plastics, building products, and medical devices (Braun et al. 2012; Ferguson et al. 2015; Kobrosly et al. 2012; Schettler 2006; Wolff et al. 2014). Finally, we calculated the sum phthalate metabolites based on their anti-androgenic activity (sum AA) by adding the DEHP metabolite concentrations of MBP, MBzP, and MiBP (Marie et al. 2015; Marsee et al. 2006).

**Table 1:** Chemicals’ limit of quantification

Chemical (parent compound)	LOQ (ng/mL)
Parabens	
Methylparaben	0.50
Ethylparaben	0.10
Propylparaben	0.50
Butylparaben	0.01
Phthalates	
MEHHP (DEHP)	0.05
MEHP (DEHP)	0.20
MEOHP (DEHP)	0.10
MECPP (DEHP)	0.05
MiBP (DiBP)	0.05
MMP (DMP)	0.20
MBzP (BBzP)	0.05
MCPP (mostly DnBP)	0.05
MEP (DEP)	0.10
MBP (DnBP)	0.05

### Untargeted metabolomics analysis

Samples were analyzed at the Metabolomics Laboratory of Roy J. Carver Biotechnology Center, University of Illinois at Urbana-Champaign, United States. Specifically, samples were initially spiked with 5 μL of the 4-Chloro-DL-phenylalanine as the internal standard (25 μg/mL) prior to processing. Processed samples were analyzed with the Q Exactive MS system (Thermo Fisher Scientific, Bremen, Germany). Software Xcalibur (version 4.1.31.9, Thermo Fisher Scientific) was used for data acquisition. The Dionex Ultimate 3000 series HPLC system (Thermo Fisher Scientific, Germering, Germany) was equipped with a degasser, an autosampler, and a binary pump. The liquid chromatography separation was performed on a Phenomenex Kinetex C18 column (4.6 × 100 mm, 2.6 mm) with mobile phase A (H2O with 0.1% formic acid) and mobile phase B (acetonitrile with 0.1% formic acid). The flow rate was 0.25 mL/min. The linear gradient was as follows: 0–3 min, 100% A; 20-30 min, 0% A; 31-36 min, 100% A. The autosampler was set to 15 °C and injection volume was 20 μL. Mass spectra were acquired under both positive (sheath gas flow rate: 45; aux gas flow rate: 11; sweep gas flow rate: 2; spray voltage: 3.5 kV; capillary temp: 250 °C; Aux gas heater temp: 415 °C) and negative electrospray ionization (sheath gas flow rate: 45; aux gas flow rate: 11; sweep gas flow rate: 2; spray voltage: -2.5 kV; capillary temp: 250 °C; Aux gas heater temp: 415 °C). The full scan mass spectrum resolution was set to 70,000 with scan range of m/z 67- m/z 1,000, and AGC target was 1E6 with a maximum injection time of 200 ms.

All liquid chromatography-mass spectrometry (LC-MS) raw data files were processed using MS-DIAL software (version 4.80) for data collection, peak detection, alignment, adduct, and identification (Tsugawa et al. 2015). The detailed parameter setting was as follows: Precursor Ion Spectrum (MS1) tolerance, 0.005 Da; Product Ion Spectrum (MS2) tolerance, 0.01 Da; minimum peak height, 100,000 amplitude; mass slice width, 0.05 Da; smoothing method, linear weighted moving average; smoothing level, 3 scans; minimum peak width, 5 scans; identification score cut off, 85%. [M-H]-, [2 M-H]- and [M + H] +, [2 M + H] + were included in adduct ion setting for negative and positive mode, respectively. Compounds were annotated by m/z and tandem mass spectrometry (MS/MS) spectra against an in-house library generated from chemical standards. In addition, they were annotated by m/z and MS/MS using the publicly available libraries MassBank of North America (MoNA), MassBank EU, and Global Natural Products Social Molecular Networking (GNPS), as well as the commercial library NIST20. Internal standards were monitored for retention time and intensity, and principal component analysis (PCA) was used for multivariate statistics and visualization of all acquired data, specifically for outlier detection. From the MS-DIAL output file, all features were removed if (sample max)/(instrument blank average) < 10. Subsequently all feature peak heights were normalized to the metabolite total ion chromatogram (mTIC). Next, positive and negative mode data were combined, each separately for known (putatively annotated) and unknown compounds. Replicates of known compounds were removed by retaining features with the highest MS-DIAL total score. Spectral matches were manually confirmed. Compounds values in peak heights (semi-quantitative) were normalized to the mTIC. Known compounds with m/z and MS2 matches were considered Metabolomics Standards Initiative (MSI) level 2 (Sumner et al. 2007), whereas unknown metabolites were considered MSI level 4.

### Statistical analyses

All statistical analyses were performed in SAS (version 9.04; SAS Institute Inc., Cary, NC, USA), with statistical significance defined as ≤ 0.05. Descriptive statistics were described by mean (± SD), median (min–max), freq (%). We assessed the normality of the data using the Shapiro-Wilk test, histograms, and Q-Q plots. For age and body weight (BW) variables that met assumptions of approximate normality, between-group differences were compared using two-sample t-tests. Differences in the proportions of sex between groups were assessed using chi-square tests. Differences in urine specific gravity (USG) and body condition scores (BCS; 1-9 ordinal scale) between groups were assessed using the Mann-Whitney U (Wilcoxon rank-sum) test.

The raw metabolomics data were log-transformed, using log(value + 1) to stabilize the variance. Then, Pareto scaling was performed by mean-centering each value and dividing by the square root of its standard deviation, standardizing the data across samples. Finally, the group variable was recoded to numeric values, and the final normalized and scaled dataset was saved for subsequent statistical analysis. Regression analyses were the primary inferential approach, while PCA, Partial Least Squares Discriminant Analysis (PLS-DA), and random forest were used as complementary multivariate methods to characterize data structure, assess group separation, and provide convergent support for key metabolomic signals, including potential non-linear patterns.

The %polynova_1way macro (Manjarin et al. 2020) was used to perform analyses of variance between the groups’ serum and urine metabolites with False Discovery Rate (FDR; Benjamini-Hochberg method) and Tukey adjustments for p-value and multiple comparisons, respectively. Each metabolite's mean log-transformed intensity (TransValue) was calculated separately for each group. The difference between the control and hyperthyroid groups means was then divided by ln(2) to yield the log2 fold change, while the fold change was computed by exponentiating the mean difference. This method ensures that a log2 fold change of 1 corresponds to a doubling of metabolite abundance between groups.

Pathway enrichment analysis was conducted across nine predefined metabolic pathway groups. For each pathway, metabolites were classified as significant or non-significant using an adjusted p-value threshold of 0.05. The number of significant and non-significant metabolites within each pathway was then summarized using PROC SQL and compared with counts from all metabolites not assigned to that pathway. Fisher’s exact tests were performed in a macro loop to evaluate enrichment of significant metabolites within each pathway group. Raw p-values were adjusted for multiple comparisons using the Benjamini-Hochberg FDR procedure, and results were merged with pathway-level counts to generate the final enrichment summary.

Unsupervised PCA was conducted to reduce data dimensionality and visualize inherent clustering or separation among samples (Chen et al. 2022). Data integrity was assessed with PROC MEANS and PROC FREQ. The dataset was recoded to binary group indicators and subjected to PCA via PROC PRINCOMP. The PCA output was further analyzed by canonical discriminant analysis, hierarchical clustering, and Pearson correlation to examine the relationship between principal component loadings and the outcome variable. Scatter plots and heatmaps were generated to visualize group separation and feature correlations.

To further dissect group differences, supervised methods were applied (Chen et al. 2022). Weighted Sub-Network Analysis (WSNA) was conducted by constructing a weighted adjacency matrix from the metabolomics data. First, pairwise Pearson correlations among metabolite features were computed and transformed using a power function (with sign preserved) to enhance stronger connections while thresholding weaker connections to zero. The resulting matrix was then reshaped into a link list of edges. Next, a topological overlap measure (TOM)-based dissimilarity matrix was derived from the adjacency matrix, normalized, and its diagonal set to zero. This dissimilarity matrix was used to perform hierarchical clustering (using Ward’s method), thereby grouping features into modules. PCA was applied for each module (or cluster) to extract the first principal component (module eigenmetabolite) as a summary measure. Finally, these eigenmetabolites were correlated with the outcome variable and evaluated using logistic regression, which allowed us to identify sub-networks of metabolites significantly associated with the phenotype of interest.

Last, PLS-DA (Chen et al. 2022) was applied to derive predictive components that maximize separation between predefined classes, thereby highlighting metabolite combinations that best discriminate hyperthyroid cats from controls. PLS-DA regression was conducted using PROC PLS on the wide-format metabolomics dataset to model group membership based on metabolite intensities. Prior to analysis, the data were transposed and merged with metadata, and the procedure was set to output variable importance measures, X-loadings, and parameter profiles. Diagnostic plots, including Variable Importance in Projection (VIP) scores and parameter profile plots, were generated and exported for further interpretation. In parallel, a random forest analysis was performed using PROC HPFOREST, with the group variable specified as the nominal target and all metabolite features as interval-level predictors. Model performance metrics, data access details, and variable importance rankings were captured and exported, allowing evaluation of the predictive performance and identification of key metabolites associated with group differences.

## Results

### Cohort characteristics

A total of 35 cats were included in the study, with 19 (11 spayed females, 8 castrated males) in the control group and 16 (10 spayed females, 6 castrated males) in the hyperthyroid group. None of the enrolled cats had consumed a low-iodine diet or received medications known to affect the hypothalamic-pituitary-thyroid axis (e.g., glucocorticosteroids, NSAIDs, antibiotics, antiseizure medications) within the 14 days preceding study enrollment. The hyperthyroid group had significantly (*p* = 0.025) lower USG [(mean ± SD): 1.035 (± 0.017); median (min-max): 1.034 (1.014-1.066)] compared to euthyroid cats [(mean ± SD): 1.049 (± 0.014); median (min–max): 1.052 (1.018-1.066)]. There were no differences in the proportions of females:males across groups (*p* = 0.78). The mean age was higher in the hyperthyroid group (13.1 ± 3.3 years) compared to the control group (10.9 ± 2.5 years; *p* = 0.03). BW was lower in the hyperthyroid group (4.06 ± 1.07 kg) than in the control group (5.57 ± 1.27 kg; *p* < 0.001). The median BCS, assessed on a 1-9 scale, was 4 (range 2-6) in the hyperthyroid group and 6 (range 5-9) in the control group, with a difference in BCS distribution between the groups (*p* = 0.001).

### Serum metabolites, urine EDC levels, and hyperthyroid status

After Tukey's adjustment for multiple comparisons, 46 serum metabolites from untargeted metabolomics and 3 urine EDCs from targeted metabolomics differed between the control and hyperthyroid groups (*p*_*adj*_ < 0.05; Online Resource 1). Serum metabolites were primarily linked to key metabolic pathways, including thyroid hormone metabolism, nitrogen metabolism, lipid metabolism, amino acid metabolism, and xenobiotic metabolism, highlighting the broad metabolic-related impacts observed in the study. Levothyroxine was markedly higher in the hyperthyroid group (fold change [FC] = 4.667, log2FC = 2.219, *p*_*adj*_ < 0.0001). Creatinine, an indicator of kidney function, was lower in the hyperthyroid group (FC = 0.669, L2FC = -0.579, *p*_*adj*_ = 0.0002). Additionally, linoleic acid, an essential omega-6 fatty acid, was lower in the hyperthyroid group (FC = 0.566, L2FC = -0.820, *p*_*adj*_ = 0.002). Conversely, 1-oleoyl-sn-glycero-3-phosphoethanolamine, a glycerophospholipid, was lower (FC = 0.359, L2FC = -1.476, *p*_*adj*_ = 0.001). Last, targeted metabolomics revealed changes in EDC levels, specifically MiBP (mono-isobutyl phthalate) ethylparaben and propylparaben, all of which were higher in the hyperthyroid group (MiBP: FC = 2.583, L2FC = 1.370, *p*_*adj*_ = 0.02; ethylparaben: FC = 3.301, L2FC = 1.723, *p*_*adj*_ = 0.03; propylparaben: FC = 2.067, L2FC = 1.048, *p*_*adj*_ = 0.03) compared the control group.

### Metabolic pathway enrichment analysis

The metabolic pathway enrichment analysis evaluated nine major metabolic pathways for differences in metabolite abundances between hyperthyroid and control cats (Table 2, Online Resource 2). No pathways reached significance after correction for multiple comparisons, although the lipid metabolism pathway (Pathway Group 3) had perturbations in 16 of 74 metabolites (raw Fisher’s *p* = 0.02, FDR-corrected *p* = 0.16).

**Table 2:** Metabolic pathway enrichment analysis across nine pre-defined pathway groups

Metabolic pathway enrichment: Fisher's exact and BH-corrected *p*-values					
Pathway name (Group number)	Pathway_sig_count	Pathway_notSig_count	Pathway_total	*p* value	
				Fisher’s Exact	BH-corrected
Carbohydrate metabolism (1)	1	21	22	0.336	0.503
Amino acid metabolism (2)	9	97	106	0.170	0.509
Lipid metabolism (3)	16	58	74	0.018	0.160
Nucleotide metabolism (4)	0	7	7	0.603	0.775
Xenobiotic/drug metabolism (5)	17	91	108	0.305	0.550
Bile acid metabolism (6)	0	5	5	1.000	1.000
Eicosanoid metabolism (7)	0	8	8	0.603	0.679
Hormone/steroid metabolism (8)	2	4	6	0.168	0.754
Other/unknown/microbial or plant secondary pathways (9)	4	49	53	0.274	0.616

**Pathway_sig_count**, **Pathway_notSig_count**, and **Pathway_total** represent the numbers of significant metabolites (adjusted *p* < 0.05), non-significant metabolites, and total metabolites assigned to each pathway group, respectively. Enrichment was assessed using Fisher’s exact tests comparing within-pathway versus outside-pathway counts, with *p*-values adjusted using the **Benjamini–Hochberg (BH)** procedure

### Principal component analysis

**Fig. 1:**
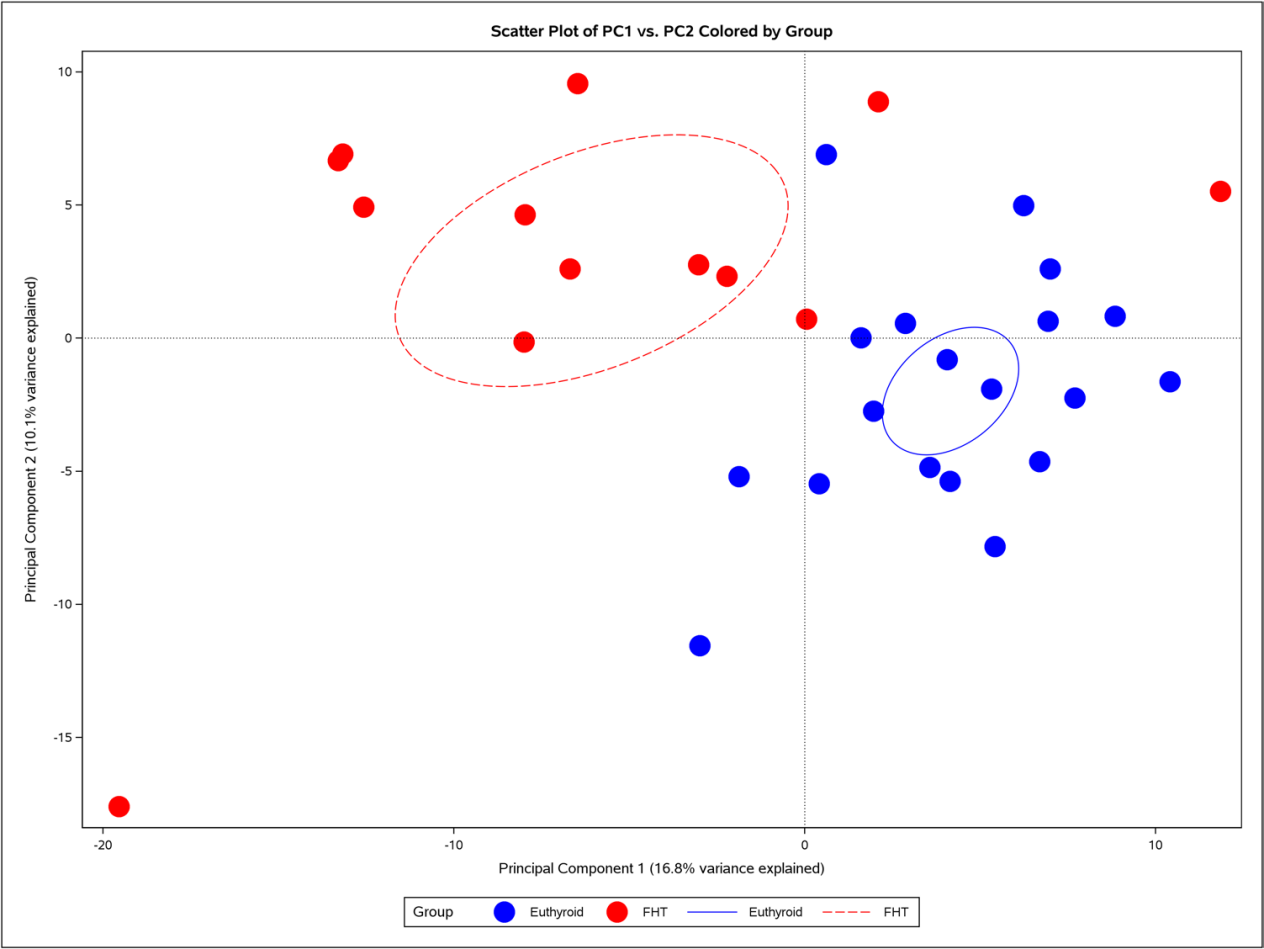
Scatter plot of the first two principal components (PC1 and PC2) derived from a principal component analysis (PCA) performed on 389 serum metabolites and urine environmental toxicants. Each point represents an individual cat, with colors indicating the outcome group: blue for control euthyroid cats and red for hyperthyroid cats. Group ellipses depict the 90% confidence region around the group mean (type = mean, α = 0.10), providing a visual summary of within-group dispersion in the reduced dimensional space. Dotted reference lines are drawn at PC1 = 0 and PC2 = 0 to mark the origin of the principal component space. The plot illustrates the distribution of observations in the reduced dimensional space, highlighting separation or clustering based on the outcome (i.e., euthyroidism vs. hyperthyroidism)

**Fig. 2:**
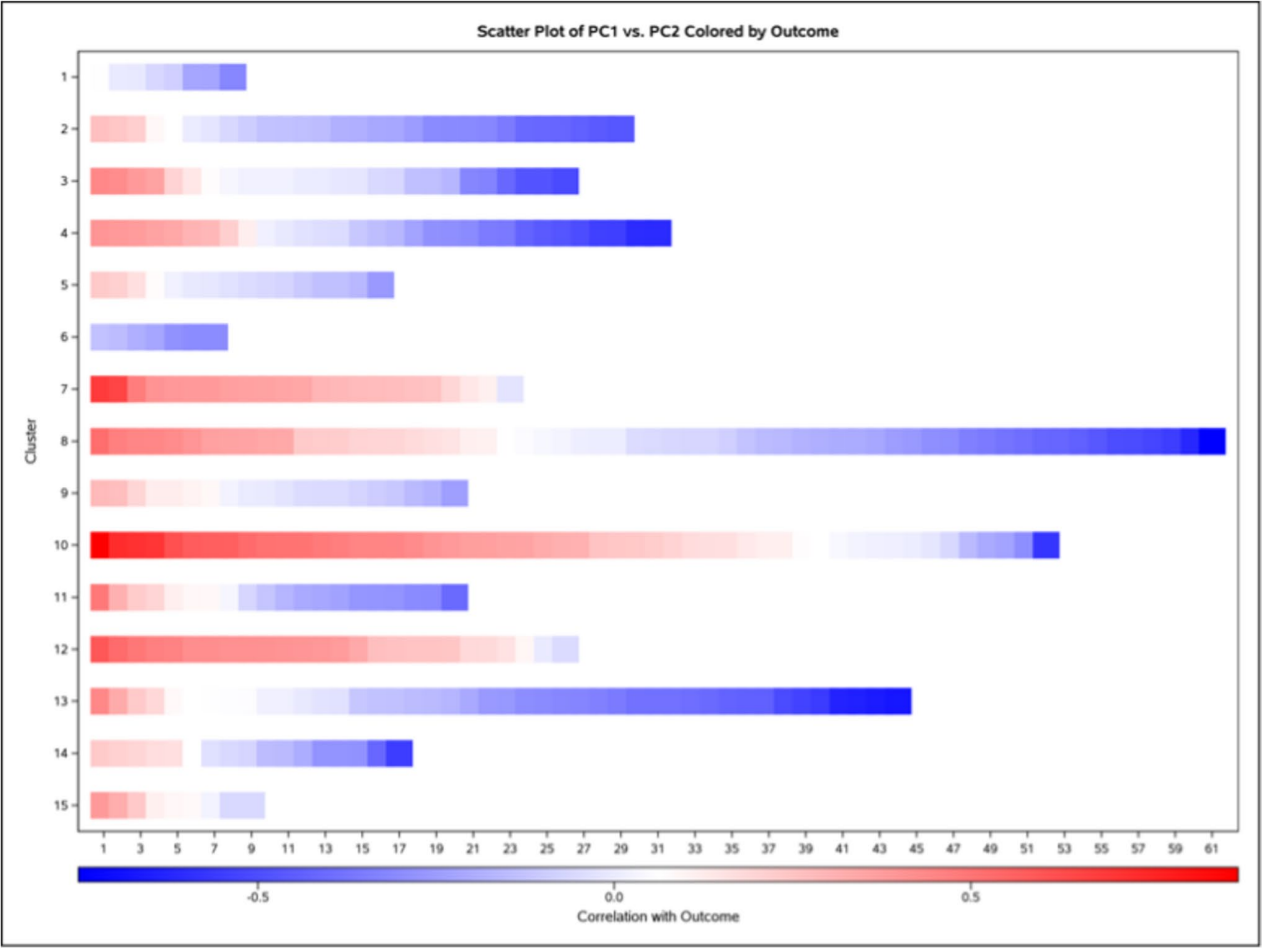
Heatmap of feature correlations with outcome across 15 clusters. The heatmap visualizes the Pearson correlation coefficients between 389 features and the outcome variable, grouped into 15 clusters based on their loadings across 21 principal components derived from PCA. The rows represent the 15 clusters (labeled 1 to 15), determined using Ward’s minimum variance clustering method on the PCA loadings. Each cluster groups features with similar patterns of contribution to the principal components. The columns represent the individual features within each cluster, ordered within each cluster by their correlation with the outcome from highest (most positive) to lowest (most negative). The X-axis is labeled with sequence numbers ranging from 1 to 61, indicating the position of each feature in this ordered list. Clusters with fewer than 61 features have empty cells beyond their last feature’s position. The color scale represents the correlation value of each feature with the outcome, using a diverging color gradient: blue for negative correlations, white for near-zero correlations, and red for positive correlations. The gradient legend at the bottom quantifies this scale

PCA was performed on a dataset comprising 389 serum metabolites and urine EDC levels, capturing 90% of the variability with the first 21 principal components. The scatter plot of the first two principal components (PC1 and PC2, Fig. 1) revealed a partial separation between the two groups predominantly across PC1. Subsequent hierarchical clustering, employing Ward’s minimum variance method on the PCA loadings, grouped the 389 features into 15 clusters (Online Resource 3) illustrated in the heatmap in Fig. 2. These clusters solely group metabolites based on their similar contribution patterns to the principal components. Post-hoc inspection revealed that these statistically derived groups correspond to biologically significant metabolic pathways. For instance, Cluster 2 is enriched with tryptophan and its derivatives, such as 5-hydroxytryptophan, a precursor in serotonin and melatonin biosynthesis. Cluster 3 includes steroid hormones, such as cortisol and cortisone, alongside synthetic opioids like butorphanol, pointing to alterations in adrenal steroid metabolism and xenobiotic processing. Cluster 8 encompasses amino acids and their derivatives, including glutamic acid, a key neurotransmitter, and creatinine, a marker of kidney function. Additionally, Cluster 10 is dominated by carnitine derivatives, such as acetyl-L-carnitine and oleoyl-L-carnitine which are involved in fatty acid transport and β-oxidation.

### Weighted subnetwork analysis

Weighted subnetwork analysis (WSNA) identified 5 distinct clusters of metabolites with shared variation patterns (Online Resource 4). The eigenmetabolite of each cluster, representing the collective expression profile of its metabolites, was correlated with hyperthyroidism using Pearson’s correlation coefficient. Logistic regression was also applied to evaluate the predictive strength of each cluster’s eigenmetabolite for hyperthyroidism, yielding odds ratios (OR) and significance levels. The findings for clusters 1–5 are detailed below, focusing on topology-informed, correlation-based modules identified by hierarchical clustering of a weighted metabolite network and summarized by cluster eigenmetabolites. **Cluster 1**: Showing a positive correlation with hyperthyroidism (r = 0.46, *p* = 0.008) and logistic regression results were Wald χ^2^ = 5.44, *p* = 0.02, odds ratio (OR) = 0.58, 95%CI: 0.37-0.92. It is enriched in tryptophan metabolism and quinoline derivatives, with metabolites such as L-kynurenine (M26) and 4-hydroxyquinoline (M94). **Cluster 2**: Exhibiting a negative correlation (r = -0.43, *p* = 0.01) and includes metabolites from amino acid metabolism and xenobiotic conjugation, such as hippuric acid (M196) and 5-hydroxyindoleacetate (M189). Logistic regression results were χ^2^ = 5.05, *p* = 0.03, OR = 1.87, 95%CI: 1.08-3.24. **Cluster 3**: Exhibiting a negative correlation (r = -0.38, *p* = 0.03) and it is associated with amino acid derivatives and synthetic compounds, including DL-isoleucine (M227) and 3-amino-4-(4-hydroxyphenyl)butyric acid (M64). Logistic regression results were χ^2^ = 3.49, *p* = 0.06, OR = 1.41, 95%CI: 0.98-2.01. **Cluster 4**: Showing a negative correlation (r = -0.40, *p* = 0.02) and it is enriched in amino acid metabolism, particularly tryptophan and its derivatives, including tryptophan (M19) and 5-hydroxytryptophan (M225). Logistic regression results were χ^2^ = 4.35, *p* = 0.04, OR = 1.41, 95%CI: 1.02-1.94. **Cluster 5**: Exhibited a negative correlation with hyperthyroidism (r = -0.58, *p* = 0.0004). It includes metabolites from lipid metabolism and xenobiotic pathways, such as linoleic acid (M153) and N-[2-(4-methoxyphenyl)ethyl]-3-phenylacrylamide (M85). Logistic regression results were χ^2^ = 7.47, *p* = 0.01, OR = 1.77, 95%CI: 1.17-2.65.

### Partial least squares discriminant analysis

**Fig. 3:**
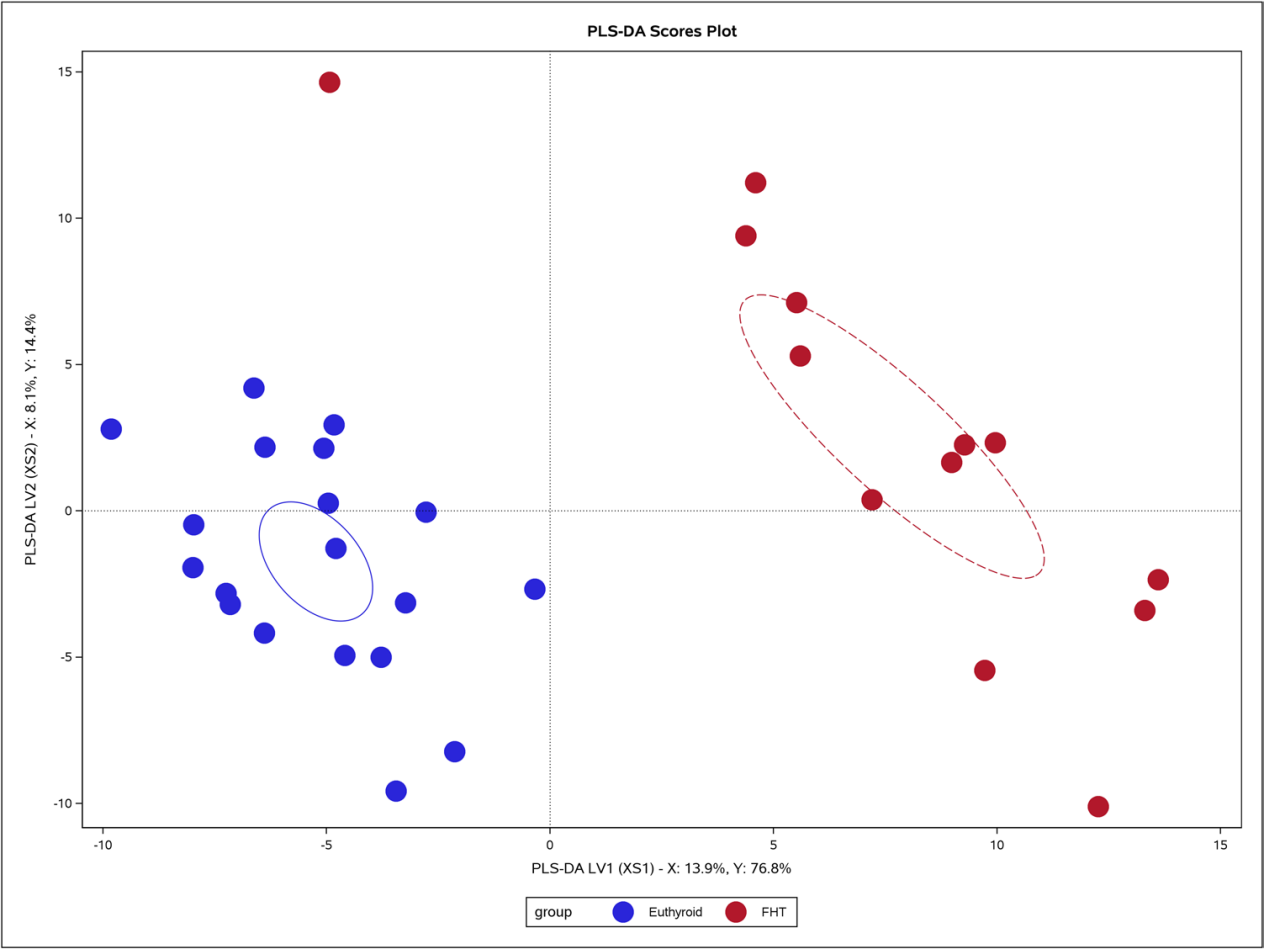
PLS-DA scores plot comparing Euthyroid and FHT samples. Scores for latent variables 1 and 2 (LV1/XS1 vs LV2/XS2) are shown for individual samples from the metabolomics dataset. Points are colored by group (blue, Euthyroid; red, FHT). Group ellipses indicate the 90% confidence region around the group mean (type = mean, α = 0.10). Dotted reference lines mark zero on each axis. LV1 accounts for 13.9% of the predictor (X) variation and 76.8% of the response (Y) variation; LV2 accounts for 8.1% of X variation and 14.4% of Y variation. Together, the first two latent variables highlight separation between Euthyroid and FHT profiles

PLS-DA modeled the relationship between 389 predictors (serum metabolites and urine EDCs) and hyperthyroidism status from 32 cats (Online Resource 5). The first two factors (Fig. 3) explained 91.1% of the variation in the response variable, with the first factor alone accounting for 76.8%. Key influential predictors, identified by high VIP scores (> 1.90) and standardized coefficients, included levothyroxine (M165) (VIP = 2.53, coefficient = 0.035), creatinine (M39) (VIP = 2.22, coefficient = -0.038), vitamin K1 (M351), found primarily in leafy green vegetables (VIP = 2.13, coefficient = 0.049), 2-hydroxybenzothiazole (M100), industrial xenobiotic (VIP = 2.09, coefficient = 0.035), 2 s-amino-4e-octadecene-1,3 s-diol (M159) long-chain base used in sphingomyelin and glycosphingolipid biosynthesis (VIP = 2.07, coefficient = 0.023), choline chloride (M20) intermediate in amino acid and lipid metabolism (VIP = 2.04, coefficient = 0.023), psychosine (M235) intermediate in sphingolipid metabolism (VIP = 1.99, coefficient = 0.02), 1-oleoyl-sn-glycero-3-phosphoethanolamine (M343) intermediate in phospholipid metabolism (VIP = 1.93, coefficient = -0.014), and l-cysteine-glutathione disulfide (M298), glutathione redox pathway (VIP = 1.90, coefficient = -0.052). These metabolites demonstrated strong associations with distinguishing euthyroid from hyperthyroid states.

### Random forest model analysis

A random forest model was developed to predict hyperthyroidism status (group) using 389 predictors (serum metabolites and urine environmental toxicants) from 32 cats (Online Resource 6). The model, configured with 100 trees and 20 variables per split, utilized an inbag fraction of 0.6 for training. It achieved a robust out-of-bag (OOB) misclassification rate of 9.4%, markedly improving upon the baseline misclassification rate of 40.6%. The most influential predictors, ranked by Gini importance, were levothyroxine (M165) (Gini = 0.024, OOB Gini = 0.019), 2 s-amino-4e-octadecene-1,3 s-diol (M159), long-chain base used in sphingomyelin and glycosphingolipid biosynthesis (Gini = 0.023, OOB Gini = 0.011), and creatinine (M39), (Gini = 0.016, OOB Gini = 0.007), underscoring their role in differentiating euthyroid from hyperthyroid states. Performance stabilized after 20-30 trees, with consistent OOB error rates, indicating robust generalization within the context of this study.

## Discussion

This study concurrently evaluated phthalate and paraben levels in association with the metabolic phenotype of FHT. By integrating metabolomic profiling with advanced multivariable and network analyses, this study provides evidence that selected EDC are associated with metabolic alterations relevant to FHT, and highlight broader implications for comparative and environmental health, as indoor cats are widely recognized as important sentinels for human exposure.

### Alterations in lipid metabolism in feline hyperthyroidism

Lipid metabolism emerged as the most perturbed pathway in hyperthyroid cats compared with euthyroid controls. Our findings extend and complement the study by Bechtold et al., who reported lower levels of free carnitine in FHT (Bechtold et al. 2024). We observed higher short-, medium-, and long-chain acylcarnitines in hyperthyroid cats that aligns with a model in which hyperthyroidism accelerates lipid turnover and energy expenditure, increasing reliance on mitochondrial β-oxidation. In this context, enhanced fatty acid flux may rapidly deplete the free carnitine pool required for mitochondrial fatty acid transport, while the acylcarnitine accumulation reflects active fatty acid conjugation to an increasingly depleted carnitine supply. Together suggesting a metabolic bottleneck wherein β-oxidation demand approaches or exceeds capacity. This interpretation is further supported by our finding of higher 3-hydroxybutyric acid levels in the hyperthyroid group, consistent with ketone overproduction when acetyl-CoA generation surpasses tricarboxylic acid cycle capacity. Last, altered levels of additional lipids and lipid derivatives (e.g., linoleic acid, glycerophospholipids, 3-hydroxydodecanoic acid, 5alpha-pregnan-3alpha-ol-11,20-dione, psychosine) further reinforce the association between thyroid hormone excess and broad disruptions in lipid homeostasis in FHT.

### Environmental endocrine disruptors and thyroid function

A novel finding of this study is that hyperthyroid cats had significantly higher urinary levels of monoisobutyl phthalate (MiBP), ethylparaben, and propylparaben. These compounds are common environmental contaminants. Specifically, MiBP is a metabolite of diisobutyl-phthalate (DiBP), a plasticizer found in flexible plastics, adhesives, and various consumer, household, and medical products. Parabens are preservatives with antimicrobial properties that are widely used in cosmetics, pharmaceuticals, and certain foods and beverages. The elevated levels of these chemicals in hyperthyroid cats suggest a possible link between environmental exposure and thyroid dysfunction in felines. This aligns with a growing body of veterinary literature implicating environmental chemicals in the etiology of FHT (Nelson et al. 2025; Nomiyama et al. 2022; Peterson 2012; Poutasse et al. 2019). For example, flame retardants and perfluorinated chemicals have been found at higher concentrations in the blood of hyperthyroid cats relative to controls (Norrgran et al. 2012, 2015; Wang et al. 2018), supporting the notion that EDCs exposure may contribute to thyroid gland pathology.

Cats are also often regarded as sentinels of human environmental exposure (Rabinowitz et al. 2008). They may encounter EDCs through inhalation of house dust and dietary ingestion, given their close contact with indoor environments, consumption of EDC-containing products, and frequent grooming behaviors (Gonkowski et al. 2025). Prior studies have shown that strictly indoor cats have higher incidences of endocrine diseases (including thyroid disorders) compared to those with outdoor access (Bree et al. 2018; Martin et al. 2000; Ohlund et al. 2017). Our findings add phthalates and parabens to the list of suspect thyroid disrupting compounds. This is concerning because phthalate metabolites are detected in essentially all tested pet cats (Karthikraj et al. 2019), and parabens have been documented in commercial cat foods and in cat urine (Karthikraj et al. 2018). As with other EDCs (Peterson 2012), exposure sources include both cat-specific pathways (e.g., pet foods, plastic toys, bowls, and litter boxes) (Braouezec et al. 2016; Karthikraj et al. 2018) and second-hand exposures from human use of everyday products such as personal care products, cleaning agents, and plastic household items. Accordingly, the mixed-source exposure scenarios warrant consideration when interpreting associations between EDC biomarkers and thyroid outcomes in cats.

EDCs can affect thyroid physiology through multiple mechanisms. Phthalates and parabens are suggested thyroid disruptors in other species, capable of interfering with the hypothalamic-pituitary-thyroid axis. For example, human epidemiological studies report associations between higher phthalate metabolite levels and both increased and decreased indicators of thyroid function, including circulating thyroid hormones (e.g., TT4) and thyroid-stimulating hormone (TSH) (Huang et al. 2017; Kim et al. 2019). Experimental data in rodents indicate that certain phthalates and parabens can suppress thyroid hormone levels, triggering feedback elevations in TSH (Azeredo et al. 2023). Butylparaben, a compound similar to propylparaben, caused dose-dependent decreases in T4 and increases in TSH in rats (Azeredo et al. 2023) consistent with an anti-thyroid effect. If cats experience a comparable impact, chronic exposure to such chemicals could initially induce a state of mild hypothyroidism or thyroid gland stress. Over time, compensatory TSH stimulation (or other growth signals) might contribute to nodular hyperplasia and adenomatous change underlying FHT (Merryman et al. 1999). This hypothesized sequence is consistent with observations in other species that chronic goitrogen exposure or iodine deficiency can lead to sustained thyroid stimulation and follicular hypertrophy/hyperplasia (Araujo-Silva et al. 2024; Colque-Caro et al. 2025; Stubner et al. 1987), and with prolonged exposure in rodent models, may promote follicular cell tumorigenesis, including thyroid adenomas (Hill et al. 1998; Kanno et al. 1992, 1990). EDC exposure may also exacerbate metabolic strain. A recent study suggests the associations between phthalate metabolite levels and lipid metabolism indicators are partly mediated by thyroid dysfunction in adult humans (Huang et al. 2022). Thus, the higher MiBP and paraben levels observed in hyperthyroid cats could reflect a cause rather than a consequence of the disease, implicating these EDCs as potential risk factors that perturb thyroid function, in cats. Future studies should examine this possibility in greater depth.

It is important to acknowledge alternative explanations as well. Hyperthyroid cats undergo metabolic changes that could influence how they absorb, distribute, or excrete chemicals. For instance, weight loss and fat mobilization in hyperthyroidism might release lipophilic toxicants stored in adipose tissue. However, phthalates and parabens are relatively short-lived (cleared within hours to days) (Koch et al. 2004; Moos et al. 2016), making it less likely that the elevated urinary MiBP and paraben reflect mobilization from fat stores unless continuous exposure is assumed. Although hyperthyroidism is typically associated with increased glomerular filtration rate, chronic kidney disease is common in senior cats and may coexist with feline hyperthyroidism; impaired renal function in these cats could influence EDC clearance.

Additionally, while hyperthyroidism is typically associated with increased glomerular filtration rate, CKD is common in senior cats and may coexist with FHT, potentially slowing EDC clearance (Boag et al. 2007; Broussard et al. 1995; Milner et al. 2006; Williams et al. 2010). As noted earlier, many hyperthyroid cats are older and indoor-only, and these conditions may increase cumulative exposure to household EDCs. Thus, while our study design cannot prove causation, the association between elevated EDCs, FHT, and the indoor environment warrants further investigation.

### Mechanistic integration and future directions

Our findings prompt several new hypotheses regarding the pathogenesis of FHT and its links to metabolism and environment. Perturbations in lipid metabolism in hyperthyroid cats may suggest that thyroid hormone excess might drive specific nutrient deficiencies or adaptive changes. For example, we hypothesize that chronic hyperthyroidism in cats may lead to functional carnitine deficiency in muscle and liver, contributing to incomplete fatty acid oxidation. This hypothesis could be tested by supplementing hyperthyroid cats (or recently treated cats) with L-carnitine and assessing improvements in their lipid metabolomic profile, weight gain, or muscle condition. A controlled trial or longitudinal study measuring carnitine levels, lipid metabolites, and clinical outcomes before and after therapy would clarify if targeting this pathway mitigates the negative metabolic impact of hyperthyroidism.

The role of EDCs in FHT warrants deeper investigation. Our results support the hypothesis that phthalates and parabens may act as etiological agents or accelerants of thyroid gland hyperplasia. A prospective longitudinal cohort study could follow healthy cats living in different environments (e.g., high-EDC vs. low-EDC households), repeatedly measure urinary EDCs levels, and monitor thyroid status to determine whether higher exposure predicts subsequent development of hyperthyroidism. Given the shared environment of cats and owners, parallel assessment of owner exposure and thyroid-related outcomes would also be valuable.

Intervention studies might also be informative, for instance, switching euthyroid cats with palpable thyroid nodules to a low-contaminant diet (free of certain packaging and additives) or improving home ventilation and dust hygiene to reduce indoor chemical loads, to determine if thyroid markers improve or disease progression slows. Although challenging, such interventions would test whether reducing EDC exposure can modulate thyroid function in cats. Last, in vitro experiments using cultured feline thyroid cells or organoids could directly test the effects of selected EDCs including phthalates and paraben on thyroid cell function.

### Study limitations

Our study has several limitations inherent to its design and the complexity of the target population. First, although we aimed to age-match our cohorts, the hyperthyroid group was significantly older than the euthyroid control group. While statistical models included age as a covariate where appropriate, we cannot fully exclude the possibility that some observed metabolic differences reflect age-related physiology rather than thyroid status alone. Second, while we excluded cats with advanced CKD (IRIS Stage 3 or higher), mild renal insufficiency is common in senior cats and was present in both groups. Differences in renal function or muscle mass, particularly muscle wasting characteristic of FHT, could influence urinary creatinine concentrations used to normalize EDC levels, potentially introducing bias. Third, this study utilized a cross-sectional design with single time-point sampling. Consequently, we could not assess the temporal relationship between EDC exposure and FHT status, nor could we assess a reverse temporal relationship in which the hyperthyroid metabolic state alters the metabolism or excretion kinetics of these environmental chemicals. Additionally, while our sample size (*n* = 35) is comparable to or larger than previous veterinary metabolomics studies, it remains relatively modest, limiting our statistical power to detect smaller effect sizes or subtler pathway perturbations. Finally, neither dietary intake nor household environmental sampling were evaluated in this study. Because commercial pet food and house dust are potential sources of EDCs, we cannot rule out their potential contribution to the heterogeneity observed in the serum metabolome and urinary EDCs profiles. Future longitudinal studies with larger, tightly matched cohorts and detailed environmental and dietary monitoring are important to validate our findings and elucidate potential roles of EDCs in FHT.

## Conclusions

In summary, while FHT has long been recognized as a metabolic disorder, this study identifies altered metabolome and associations with elevated EDCs burdens in affected cats. The association between hyperthyroidism and higher EDC levels may suggest that environmental pollutants may contribute to disease pathogenesis, positioning cats as sentinels for household chemical exposures. Additionally, untargeted metabolomics proved valuable for characterizing metabolomic fingerprints useful for generating testable hypotheses for future studies.

## Supplementary Information

Below is the link to the electronic supplementary material.

Supplementary file1 (XLSX 37.7 KB)

Supplementary file2 (XLSX 83 KB)

Supplementary file3 (XLSX 40 KB)

Supplementary file4 (XLSX 22 KB)

Supplementary file5 (XLSX 30 KB)

Supplementary file6 (XLSX 30 KB)

## Data Availability

No datasets were generated or analysed during the current study.

## Abbreviations

AA: Anti-Androgenic Activity
BBzP: Butyl Benzyl Phthalate
BCS: Body Condition Score
BW: Body Weight
CI: Confidence Interval
CKD: Chronic Kidney Disease
DBP: Di-n-Butyl Phthalate
DEHP: Di(2-ethylhexyl) Phthalate
DEP: Diethyl Phthalate
DiBP: Di-isobutyl Phthalate
DMP: Dimethyl Phthalate
EDCs: Endocrine-Disrupting Chemicals
FC: Fold Change
FDR: False Discovery Rate
FHT: Feline Hyperthyroidism
HPLC: High-Performance Liquid Chromatography
HT: Hyperthyroidism
IACUC: Institutional Animal Care and Use Committee
L2FC: Log2 Fold Change
LC-MS: Liquid Chromatography-Mass Spectrometry
LC-MS/MS: Liquid Chromatography-Tandem Mass Spectrometry
LDL: Low-Density Lipoprotein
LOQ: Limit of Quantitation
MBP: Mono-n-Butyl Phthalate
MBzP: Monobenzyl Phthalate
MCPP: Mono(3-Carboxypropyl) Phthalate
MECPP: Mono(2-ethyl-5-carboxypentyl) Phthalate
MEHHP: Mono(2-ethyl-5-hydroxyhexyl) Phthalate
MEHP: Mono(2-ethylhexyl) Phthalate
MEP: Monoethyl Phthalate
MEOHP: Mono(2-ethyl-5-oxohexyl) Phthalate
MiBP: Mono-isobutyl Phthalate
MMP: Monomethyl Phthalate
MS1: Precursor Ion Spectrum
MS2: Product Ion Spectrum
MSI: Metabolomics Standards Initiative
MS/MS: Tandem Mass Spectrometry
mTIC: Metabolite Total Ion Chromatogram
MW: Molecular Weight
OOB: Out-of-Bag
PBDEs: Polybrominated Diphenyl Ethers
PC1: Principal Component 1
PC2: Principal Component 2
PCA: Principal Component Analysis
PCP: Personal Care Products
PLS-DA: Partial Least Squares Discriminant Analysis
PROC: Procedure (SAS)
SD: Standard Deviation
TT4: Total Thyroxine
TOM: Topological Overlap Measure
TSH: Thyroid-Stimulating Hormone
USG: Urine Specific Gravity
VIP: Variable Importance in Projection
VTH: Veterinary Teaching Hospital
WGCNA: Weighted Correlation Network Analysis
WSNA: Weighted Subnetwork Analysis
